# Effects of drinking water supplementation with *Lactobacillus reuteri*, and a mixture of reuterin and microcin J25 on the growth performance, caecal microbiota and selected metabolites of broiler chickens

**DOI:** 10.1186/s40104-022-00683-6

**Published:** 2022-03-05

**Authors:** Liya Zhang, Laila Ben Said, Nadège Hervé, Séverine Zirah, Moussa Sory Diarra, Ismail Fliss

**Affiliations:** 1 Institute of Nutrition and Functional Foods, Université Laval, QC, Québec Canada; 2 Sollio Agriculture, Montréal, QC Canada; 3 Molécules de Communication et Adaptation des Microorganismes, Muséum National d’Histoire Naturelle, Paris, France; 4 Guelph Research and Development Centre, Agriculture and Agri-Food Canada, Guelph, ON Canada

**Keywords:** Broiler chicken, *Lactobacillus reuteri*, Metabolome, Microbiome, Microcin J25, Reuterin, SCFA

## Abstract

**Background:**

Since the overuse of antibiotics in animal production has led to a selection of antibiotic-resistant pathogens that affect humans and animals as well. Scientists are therefore searching for novel natural alternatives to antibiotics. In this study *Lactobacillus reuteri* and a combination of reuterin and microcin J25 (RJ) were evaluated as promoters of growth and modulators of the cecal microbiota and metabolite profiles in broiler chickens. One-day-old Cobb 500 male broilers were distributed to 8 treatments: negative control (without antibiotic), positive control (bacitracin), three concentrations of RJ and three doses of *L. reuteri* plus glycerol. The birds (2176, 34 per pen, 8 pens per treatment) were reared for 35 d.

**Results:**

The body weight of the bacitracin and 5 mmol/L reuterin combined with 0.08 μmol/L microcin J25 (10RJ) treatment group was significantly higher than that of the negative control group (*P* < 0.05). *L. reuteri* had no significant effect on broiler growth. MiSeq high-throughput sequencing of 16S rRNA showed clustering of cecal microbial operational taxonomic unit diversity according to treatment. The influence of bacitracin and 10RJ on bacterial community overall structure was similar. They promoted Ruminococcaceae*,* Lachnospiraceae and Lactobacillaceae*,* increased the relative abundance of *Faecalibacterium* and decreased the abundance of *Bacteroides* and *Alistipes*, while the negative control condition favored Bacteroidaceae and Rikenellaceae. Furthermore, 10RJ increased the concentration of short-chain fatty acid in the cecum and changed the metabolome overall.

**Conclusions:**

These overall suggest that 10RJ can promote a host-friendly gut environment by changing the cecal microbiome and metabolome. This combination of natural antimicrobial agents in the drinking water had a positive effect on broiler growth and may be suitable as an alternative to antibiotic growth promoters.

**Supplementary Information:**

The online version contains supplementary material available at 10.1186/s40104-022-00683-6.

## Introduction

The intestinal microbial community plays an important role in promoting general and immunological health and improving feed digestion and nutrient absorption and thus improving growth performance in food-producing animals [1]. Antibiotics are antimicrobial agents that may be used to kill pathogenic microbes in the intestine, lessen the burden on the immune system, optimize the digestive system, and improve the growth performance [2]. Antibiotics have been used extensively in poultry production to maintain animal health and to improve the growth of animals. They can decrease utilization of nutrients by the gut microbiota and thereby keep these nutrients available for absorption and utilization by the body [3]. However, the continuous use of antibiotics in animal production has led to a selection of antibiotic-resistant pathogens that affect humans and animals as well. Scientists are therefore searching continually for novel natural alternatives to antibiotic growth promoters.

Several alternatives have been considered, such as probiotics, which are defined as live microorganisms that confer a health benefit on the host [4]. Their advantageous traits include surviving and germinating in the gastrointestinal tract as well as secreting protease, amylase and lipase [1]. *Lactobacillus reuteri,* although used as probiotic for more than 10 years in health products [5], has been studied somewhat less in the poultry industry. Under suitable culture conditions, *L. reuteri* converts glycerol to reuterin, a water-soluble potent inhibitor of Gram-positive and Gram-negative bacteria over a wide range of pH and not affected by hydrolytic enzymes [6]. Believed to play a role in the probiotic effect of *L. reuteri*, reuterin can be synthesized in the colon when sufficient amounts of glycerol are available [5]. Some *L. reuteri* have been found to inhibit *Listeria monocytogenes* and *Escherichia coli* O157:H7 in cheese [7], sausage [8] and ground pork [9] through the effect of reuterin. Meanwhile, the poultry sector has completely ignored reuterin.

Bacteriocins are proteinaceous molecules that possess bacteriostatic or bactericidal activities against relatively narrow spectra of taxa generally related closely to the producing strain by forming pores in cell membranes and/or inhibiting cell wall synthesis [10]. Produced by *E. coli*, microcin J25 is bactericidal to several Gram-negative food-borne pathogens including *E. coli* and *Salmonella* [11]. Its peculiar lasso structure makes it highly resistant to thermal denaturation and stressful gastrointestinal conditions as previously shown [12]. Because of these features, microcin J25 has been investigated as a replacement for conventional antibiotic growth promoters in broiler chicken production [13]. To the best of our knowledge, the combined effect of reuterin and microcin J25 on broiler chicken growth has not been studied. Combinations may make each antimicrobial compound effective at a lower concentration and the emergence of resistant variants less likely [14, 15]. In our previous study [16], compared to using them alone, the combination of reuterin and microcin J25 (RJ) at lower concentration has already been proved to be synergistic for reducing counts of *Salmonella enterica* on chicken carcasses.

High-throughput sequencing technologies have contributed much to the current understanding of intestinal microbial ecology. An important section in the gastrointestinal tract is the cecum, in which most fermentation occurs, with a strong influence on intestinal health and animal nutrition. Intestinal fermentation products such as short-chain fatty acids (SCFA) represent an essential contribution of cecal microorganisms to host metabolism and have positive effects on gut health [17]. The activities of *Ruminococcus, Streptococcus, Faecalibacterium, Lactobacillus* and *Clostridium* clusters IV, XIVa and XIVb increase host assimilation of complex substrates by converting them partly to SCFA including butyrate [17]. Metabolomics is essentially the comprehensive characterization of the metabolites of the biosystem.

For the present study, we hypothesized that natural antimicrobial combination could have a positive effect on the gut microbiota and metabolome, thereby supporting broiler chicken growth performance equivalent to that obtained using antibiotics. We therefore evaluated RJ as well as *L. reuteri*, in terms of growth performance, cecal microbial community composition, and changes to metabolite profiles.

## Materials and methods

The study was conducted at the Sollio Agriculture Research Station (St-Jean-Baptiste, QC, Canada). The experimental protocol (No. 2019054–1) including the management and care of animals was reviewed and approved by Animal Protection Committee of Université Laval.

The chemical reagents, unless otherwise stated, were purchased from MilliporeSigma (St. Louis, MO, USA).

### Reuterin and microcin J25 production

A two-step fermentation process was used to produce reuterin as described previously [18]. *L. reuteri* was cultured in 20 L of MRS medium (Nutri-Bact, Terrebonne, QC, Canada) and incubated anaerobically overnight at 37 °C. The cells were harvested by centrifugation at 1500×*g* for 10 min at 20 °C, washed with potassium phosphate buffer (0.1 mol/L, pH 7.0) and resuspended in 2 L of sterile water containing glycerol (300 mmol/L). After 2 h, the suspension was centrifuged at 15,000×*g* for 10 min at 4 °C and micro-filtered (0.2 μm, Millipore, Darmstadt, Germany). Compounds were identified and quantified using a Coregel ION300 column (7.8 × 300 mm, Cobert Associates, Inc., Saint Louis, MO, USA) with 10 mmol/L H_2_SO_4_ at 40 °C as eluent at 0.4 mL/min in an HP Agilent 1100 high-performance liquid chromatography (HPLC) with a refractive index detector (Agilent Technologies, Santa Clara, CA, USA). The reuterin solution was stored at − 20 °C until use.

Microcin J25 was produced by *E. coli* MC4100 cultured in minimal medium M63 as described previously [19–21]. The supernatant was purified by disposable solid-phase extraction using a Sep-Pak C18 35 cc vac cartridge (Waters, Milford, MA, USA) at 4 °C. The peptide was separated by hydrophobicity using acetonitrile/water (0 and 20% v/v) with 0.1% HCl and eluted with acetonitrile/water (30% v/v) containing 0.1% HCl at a flow rate of 10 mL/min. Acetonitrile was removed using a Rotavapor R-215 (BÜCHI Labortechnik AG, Flawil, Switzerland) and the aqueous portion was micro-filtered (0.2 μm, Merck KGaA, Darmstadt, Germany). Microcin was quantified by HPLC (HP1100 with a C18 column, Gemini® 5 μm NX-C18 110 Å, 250 × 4.6 mm, Phenomenex, Torrance, CA, USA).

In our previous study, the minimum inhibitory concentration of RJ on *S. enterica* was determined to be 0.5 mmol/L (reuterin) and 0.008 μmol/L (microcin J25), respectively [16]. Concentrations of 1, 5, and 10 times the minimum inhibitory concentration of RJ were used in the present study.

### Bacterial strains

*L. reuteri* isolates C1–8, C1–14 and C1–18 from broiler chicken intestine (unpublished) were cultured overnight in MRS media (Nutri-Bact, Terrebonne, QC, Canada) at 37 °C. The cells were centrifuged (Heraeus Multifuge 1S-R, Hanau, Germany) at 5000×*g* for 10 min at 4 °C, the culture broth was removed, and the pellet was suspended in an equal volume of 20% skim milk (Hardy diagnostics, Santa Maria, CA, USA). The three strains were blended after freeze-drying (Labconco Freeze Dryer, Kansas City, MO, USA).

### Chickens and experimental design

One-day-old Cobb 500 broiler chickens (*n* = 2176, male) were distributed randomly into 8 treatment groups. Each treatment consisted of 8 replicate pens with 34 birds each (2.25 m^2^, density of 15 birds/m^2^) and reared for 35 d. All birds were vaccinated against coccidiosis in the hatchery. All groups received a basal diet (See Additional file 1: Supplementary Table 1) containing phytase and xylanase for the starter, grower, and finisher periods. The feeds were produced at the Sollio Agriculture facility (Joliette, QC, Canada) with wheat, corn, soybean meal, extruded ground soy and canola meal, and the composition was analyzed by Eurofins EnvironeX laboratory (Québec, QC, Canada). No additional anticoccidials or additives were administrated to the birds throughout the trial. The starter and grower diet were in the form of crumble, while the finisher diet was in pellet form. Feed and fresh water were available ad libitum*.* Temperature and lighting were controlled as recommended in the industry (See Additional file 1: Supplementary Table 2 and Table 3). A data logger was placed in the middle of the room to record temperature and moisture content every hour (See Additional file 1: Supplementary Fig. 1).

The experimental treatments were consisted of 55 mg/kg antibiotic bacitracin (positive control, PC), no antibiotic (negative control, NC), 0.5 mmol/L reuterin + 0.008 μmol/L microcin J25 (1RJ), 2.5 mmol/L reuterin + 0.04 μmol/L microcin J25 (5RJ), 5 mmol/L reuterin + 0.08 μmol/L microcin J25 (10RJ), *L. reuteri* (10^6^ CFU/mL) + 300 mmol/L glycerol (E6Lr), *L. reuteri* (10^7^ CFU/mL) + 300 mmol/L glycerol (E7Lr), and *L. reuteri* (10^8^ CFU/mL) + 300 mmol/L glycerol (E8Lr). The antibiotic was added to the feed according to the commercial usage. The alternative antimicrobial solutions were added to the drinking water in the manual drinker. The treatments were received from d 0 to 14. Since manual drinkers were used, unexpected volume of water was occasionally wasted due to chicken’s moving on the drinker. 15% and 30% of the initial volume of water was added in all groups on d 3 and d 4–6 respectively to compensate for observed waste and reduce possible unwanted water stress.

### Broiler growth performance

Feed intake and feed conversion ratio were recorded per pen for each growth phase. Body weight and average daily gain were measured on d 0, 7, 10, 22 and 35 for each pen. Mortality was recorded daily.

### Cecal microbial DNA extraction and analysis

On the d 21, two broiler chickens per pen were selected randomly and euthanized by cervical dislocation. The cecal digesta were collected in sterile plastic tubes and the two from each pen were combined in equal proportions. The resulting 64 samples (8 replicate pens × 8 treatment groups) were stored at –20 °C until analysis of microbiota and microbial metabolites, which was completed within 2 weeks after sample collection.

Microbial total genomic DNA was extracted from 250 mg of cecal sample using the QIAamp PowerFecal DNA Kit (Qiagen, Hilden, Germany) according to the manufacturer’s instructions. DNA quality and concentration were assessed using a NanoDrop 1000 spectrophotometer (Thermo Scientific, Wilmington, DE, USA). The 16S rRNA V3–V4 region was amplified and sequenced at the Université Laval Genomic Analysis Platform-IBIS using Illumina MiSeq paired-end technology. Sequences were analyzed in Ubuntu terminal using the UPARSE method [22], merging raw reads to 430–480 bp length, filtering with the maximum expected error threshold at 1.0, dereplicating and mapping reads into operational taxonomic units (OTUs). The “sintax” command was used to predict taxonomy for each OTU using the RDP training set v16 database [23].

### Analysis of the cecal metabolome

Thawed digesta were homogenized in a Bead Ruptor 12 Homogenizer (Omni International, Kennesaw, USA) using 3 mm glass beads as previously described [24] with some modifications. Briefly, samples weighing 100 mg were suspended in 1 mL of 70% isopropanol and agitated at high speed for 30 s twice with a 60 s pause. Dry weight was determined using 250 μL of homogenate in a vacuum centrifuge overnight (SPD131DDA SpeedVac™ Concentrator, Thermo Scientific, Asheville, NC, USA). The remaining homogenate was diluted to 5 mg/mL, stored at − 80 °C and transported on dry ice to the metabolomic analysis lab (Muséum national d’Histoire naturelle, Paris, France).

An aliquot of the homogenate was centrifuged, and the clear supernatant was derivatized with 3-nitrophenylhydrazone (3-NPH) as described by Liebisch et al. [24] with slight modifications. Briefly, an aqueous internal standard (50 μL) containing [D_3_]-acetic acid and [D_5_]-propionic acid (100 μg/mL each) and [D_7_]-butyric acid (500 μg/mL) was mixed with 50 μL of supernatant. The 3-nitrophenylhydrazine hydrochloride (200 mmol/L, 20 μL) and N-(3-dimethylaminopropyl)-N′-ethylcarbodiimide hydrochloride (120 mmol/L, 20 μL) were mixed in for 30 min at 40 °C. The reaction was quenched with 500 μL of 0.1% formic acid.

A LC (Ultimate 3000 RSLC, Thermo Scientific, Asheville, NC, USA) coupled to a high-resolution electrospray-ionization quadrupole time-of-flight mass spectrometer (Maxis II ETD, Bruker Daltonics, Billerica, MA, USA) was used. Metabolite separation was achieved on an Acclaim RSLC Polar Advantage II column (2.2 μm, 2.1 × 100 mm, 120 Å, Thermo Scientific, Asheville, NC, USA) at a flow rate of 300 μL/min using gradients of solvent A (ultra-pure water/0.1% formic acid) and solvent B (HPLC-MS grade acetonitrile/0.08% formic acid). For untargeted metabolites, the gradient run time was 17.5 min: 5 min at 2% B followed by an increase to 10% B for 7 min, then an increase to 80% B for 0.5 min, and to 100% for 1 min, then down to 2% B for 4 min. LC-MS and data-dependant LC-MS/MS data were acquired in positive ion mode in the *m/z* = 100–1300 range. Analysis quality control was ensured by injecting a mixture of all samples every 10 runs to monitor separation and check for cross-contamination. For SCFA, the gradient run time was 7 min: 3 min at 20% B followed by a linear increase to 50% B for 0.5 min, increase to 100% B for 1 min and down to 20% B for 2.5 min. LC-MS data were acquired in negative ion mode in the *m/z* = 100–250 range. The retention times and *m/z* ratios of the SCFA 3-NPH derivatives and corresponding stable deuterated internal standards are provided (See Additional file 1: Supplementary Table 4). Assignment of *m/z* values to the SCFA derivatives and deuterated standards were verified by LC-MS/MS.

### Statistical analysis

The data of growth performance was presented as least square means. The individual pens were used as experimental units (eight pens/treatment group) for all statistical analyses. The evaluation of the experimental treatments was carried out through performance analysis (body weight, average daily gain, feed intake, feed conversion ratio) according to a complete randomized block design with the mixed procedure on R (lme function). Both normal distribution and homogeneity of variance were validated graphically. In case of significant effect, the average comparison was carried out using a Tukey’s test. Mortality proportions were analyzed using binominal logistic regression (glmer function on R). If a significant treatment effect was observed with the frequency analysis, a 2 by 2 treatment comparison was performed to determine the differences using a Tukey’s test. Differences were regarded significant at *P* < 0.05.

Genomic data were visualized and analyzed using the ampvis2 R-package in RStudio and the web-based tool MicrobiomeAnalyst. The Shannon diversity index was analyzed to compare further the treatment effects. Beta diversity was evaluated by principal coordinate analysis (PCoA) based on the Bray-Curtis distance [25]. To obtain species-level classification, sequences of dominating OTUs were analyzed in the EzBioCloud database [26]. PERMANOVA analysis, using a permutation method under a reduced model, was used to study the significant differences (*P* < 0.05) between the different groups. Correlation network analysis was based on the SparCC algorithm, with the permutation set at 100, *P* value at 0.05 and a correlation threshold of 0.3 at the genus level. Nodes indicated the genus and were colored based on their abundance for each treatment.

## Results and discussion

### Growth performance

The effects of the experimental treatments on body weight, feed intake, average daily gain, feed conversion ratio, and mortality of broiler chickens are shown in Table 1. Compared to NC, the groups subjected to an antibiotic treatment and to 10RJ showed significant increases in feed intake, body weight and average daily gain (*P* < 0.05). None of the treatments tested had a significant effect on the feed conversion ratio relative to NC. Mortality in the NC group did not differ statistically (*P* > 0.05) from any other group, except for the group receiving the E6Lr. No statistical differences were found between the antibiotic-receiving group and the 10RJ group throughout the study. The 1RJ, 5RJ and *L. reuteri* treatments did not show a significant (*P >* 0.05) effect on the growth performance of the chickens during the experiment period (Table 1).

**Table 1:** Effect of different doses of *Lactobacillus reuteri*, and a mixture of reuterin and microcin J25 on growth performance of broiler chickens from d 0 to 35 of experiment

Variables	Treatments									
	PC	NC	1RJ	5RJ	10RJ	E6Lr	E7Lr	E8Lr	*SEM*	*P*-value
***Feed intake, kg***										
d 0 to d 10	0.29^a^	0.27^bc^	0.28^ab^	0.28^ab^	0.29^a^	0.26^cd^	0.26^d^	0.26^cd^	0.004	<.0001
d 11 to d 22	1.18^a^	1.09^c^	1.12^abc^	1.13^abc^	1.15^ab^	1.12^bc^	1.09^c^	1.09^c^	0.013	<.0001
d 0 to d 22	1.47^a^	1.36^cd^	1.40^bcd^	1.41^abc^	1.44^ab^	1.38^bcd^	1.34^d^	1.35^cd^	0.015	<.0001
d 23 to d 35	2.41^a^	2.28^b^	2.37^ab^	2.32^ab^	2.40^a^	2.33^ab^	2.28^b^	2.35^ab^	0.027	0.001
d 0 to d 35	3.87^a^	3.64^c^	3.77^abc^	3.73^bc^	3.83^ab^	3.71^bc^	3.63^c^	3.70^bc^	0.037	<.0001
***Body weight, kg***										
d 0	0.05	0.05	0.05	0.05	0.05	0.05	0.05	0.05	0.0002	0.350
d 7	0.20^a^	0.19^abc^	0.20^ab^	0.19^abc^	0.20^a^	0.18^c^	0.19^bc^	0.19^c^	0.004	<.0001
d 10	0.31^a^	0.30^c^	0.30^bc^	0.30^bc^	0.31^ab^	0.30^bc^	0.30^c^	0.30^abc^	0.004	0.0002
d 22	1.19^a^	1.12^c^	1.13^bc^	1.15^abc^	1.17^ab^	1.13^c^	1.12^c^	1.13^c^	0.017	<.0001
d 35	2.82^a^	2.65^c^	2.73^abc^	2.68^bc^	2.77^ab^	2.70^bc^	2.67^bc^	2.70^bc^	0.026	0.0001
***Average daily gain, g***										
d 0 to d 7	22.3^a^	21.0^bcd^	21.4^abc^	21.3^abcd^	22.2^ab^	19.5^d^	20.2^cd^	20.2^d^	0.53	<.0001
d 8 to d 10	37.8^abc^	34.5^c^	35.4^bc^	36.0^bc^	37.2^abc^	39.9^a^	36.8^abc^	38.7^ab^	0.84	0.0001
d 0 to d 10	27.0^a^	25.1^c^	25.6^bc^	25.7^abc^	26.7^ab^	25.6^abc^	25.2^cd^	25.7^abc^	0.35	0.0001
d 11 to d 22	72.7^a^	68.9^b^	68.9^b^	70.7^ab^	71.4^ab^	68.9^b^	68.4^b^	68.5^b^	0.97	0.001
d 0 to d 22	51.9^a^	49.0^c^	49.3^bc^	50.3^abc^	51.1^ab^	49.2^bc^	48.8^c^	49.1^bc^	0.63	<.0001
d 23 to d 35	125.2^a^	117.6^b^	123.0^ab^	117.3^b^	123.3^ab^	120.9^ab^	119.3^ab^	121.0^ab^	1.47	0.003
d 0 to d 35	79.1^a^	74.5^c^	76.7^abc^	75.2^bc^	77.9^ab^	75.9^bc^	75.0^bc^	75.8^bc^	0.74	0.0001
***Feed conversion ratio, g:g***										
d 0 to d 10	1.10^b^	1.12^b^	1.12^b^	1.12^b^	1.10^b^	1.04^a^	1.06^a^	1.05^a^	0.006	<.0001
d 11 to d 22	1.50^a^	1.54^b^	1.52^ab^	1.51^ab^	1.51^ab^	1.49^a^	1.49^a^	1.51^ab^	0.010	0.012
d 0 to d 22	1.39^abc^	1.42^d^	1.41^cd^	1.40^bcd^	1.40^abcd^	1.37^a^	1.38^ab^	1.39^abc^	0.007	<.0001
d 23 to d 35	1.50	1.50	1.51	1.53	1.52	1.50	1.50	1.50	0.013	0.088
d 0 to d 35	1.45^abc^	1.47^abc^	1.47^abc^	1.47^c^	1.47^bc^	1.45^ab^	1.45^a^	1.45^abc^	0.005	0.001
***Mortality, %***										
d 0 to d 7	5.5	6.6	6.6	4.0	4.4	4.8	7.7	7.7	1.6	0.425
d 8 to d 10	1.0	2.0	2.0	2.3	1.3	1.0	1.6	1.3	1.0	0.856
d 11 to d 22	3.7^ab^	9.2^b^	4.0^ab^	6.6^ab^	5.2^ab^	2.6^a^	5.2^ab^	5.9^ab^	1.8	0.038
d 23 to d 35	2.4	1.7	0.7	1.0	2.0	1.7	1.4	1.4	1.0	0.789
d 0 to d 35	12.9^ab^	19.9^b^	13.6^ab^	14.3^ab^	13.2^ab^	10.3^a^	16.2^ab^	16.5^ab^	2.4	0.045

^a,b,c,d^ means in each raw with different superscripts are significantly different (*P* < 0.05). *SEM* = standard error of means

PC = antibiotic bacitracin, NC = without antibiotic, 1RJ = 0.5 mmol/L reuterin and 0.008 μmol/L J25, 5RJ = 2.5 mmol/L reuterin and 0.04 μmol/L J25, 10RJ = 5 mmol/L reuterin and 0.08 μmol/L J25, E6Lr = *L. reuteri* (10^6^ CFU/mL) plus 300 mmol/L glycerol, E7Lr = *L. reuteri* (10^7^ CFU/mL) plus 300 mmol/L glycerol, E8Lr = *L. reuteri* (10^8^ CFU/mL) plus 300 mmol/L glycerol. (*n* = 8 replicates/treatment)

The positive effects of antibiotics and probiotics on broiler chicken growth have been shown previously [27–29]. However, there is less literature on the effects of reuterin and microcin J25 on the growth of broilers. Only one study showed that dietary supplemented microcin J25 at 0.5 mg/kg and 1 mg/kg (0.24 and 0.48 μmol/L) appeared to promote growth performance compared to a pathogen-challenged group [13]. The higher concentration of 2.0 mg/kg (0.95 μmol/L) of microcin J25 was indicated as optimal dosage for pig growth and gut health [30]. In the present study, by using a lower concentration (5 mmol/L reuterin + 0.08 μmol/L microcin J25) than in other research, the combination added to the drinking water improved feed intake, body weight and average daily gain of broilers (*P* < 0.05), with the same efficacy as the antibiotic. Although feed conversion ratio and mortality were not affected by the treatments, better feed intake and average daily gain in broilers fed antibiotics or given the 10RJ led to greater body weight during the experiment period. *Lactobacillus-*based probiotics were previously reported to be an effective alternative to antibiotic growth promoters in poultry production [31]. However, we did not observe significant effects of *L. reuteri* at different doses. Better results might be obtained with higher doses or mixture of different species rather than a single species. Other factors including diet, stress and management may also affect effectiveness. Studies using reuterin, microcin, and *L. reuteri* remain limited.

### Microbial composition of cecal digesta

**Fig. 1:**
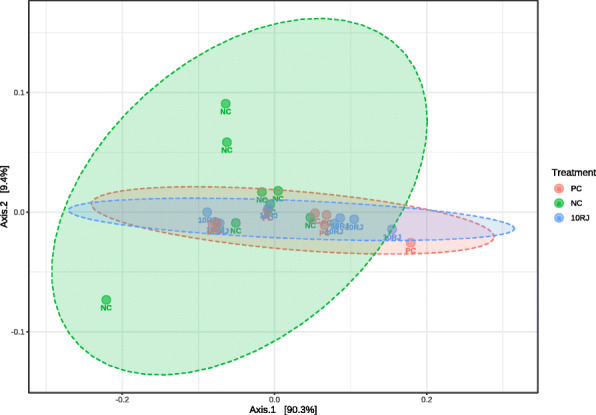
Principal coordinates analysis (PCoA) of bacterial communities in the cecal digesta of broilers (based on the Bray distance). PC = basal diet with antibiotic bacitracin, NC = basal diet without antibiotic, 10RJ = basal diet with 5 mmol/L reuterin and 0.08 μmol/L J25 in drinking water. *P* < 0.18

**Fig. 2:**
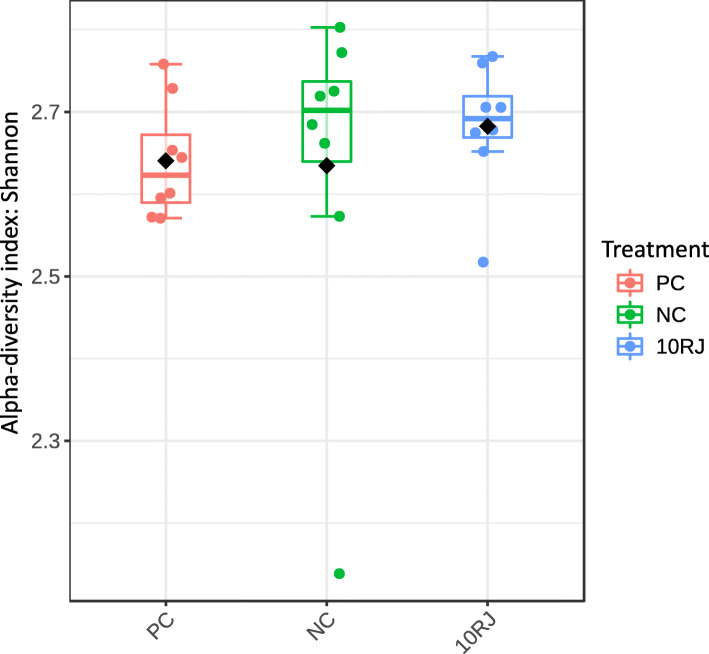
Shannon diversity index for the three treatments. PC = basal diet with antibiotic bacitracin, NC = basal diet without antibiotic, 10RJ = basal diet with 5 mmol/L reuterin and 0.08 μmol/L J25 in drinking water. *P* > 0.05

The 16S rRNA MiSeq sequences from the 64 samples of broiler cecal microbiota were used for subsequent analyses. PCoA with Bray distance indicated that the NC group was distinct from the antibiotic and 10RJ groups (Fig. 1), but this was not the case for any *L. reuteri* treatment group. There was an apparent clustering of cecal microbiota for the antibiotic and 10RJ groups. The Shannon diversity index did not show significant statistical differences between the treatments, which confirms species abundance as the driving factor for the variations (Fig. 2). Since the 1RJ, 5RJ and *L. reuteri* treatment groups showed neither significant differences from the NC group nor a positive growth-promoting effect, further analysis for treatment comparison was focused on the 10RJ treatment group.

**Fig. 3:**
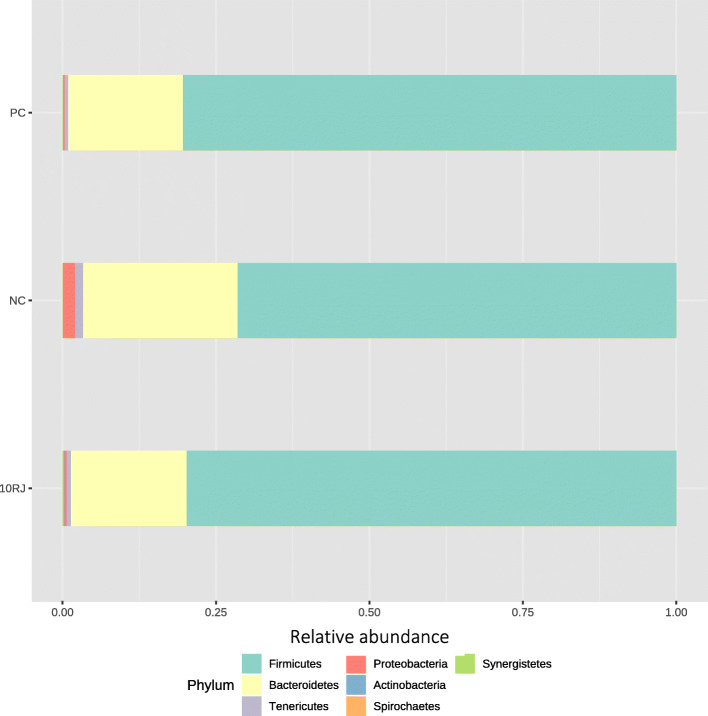
Relative abundance of the broilers’ caecal microbiota in level phylum. Each mean represents eight samples. PC = basal diet with antibiotic bacitracin, NC = basal diet without antibiotic, 10RJ = basal diet with 5 mmol/L reuterin and 0.08 μmol/L J25 in drinking water

The top five phyla identified were Firmicutes, Bacteroidetes, Tenericutes, Proteobacteria, Actinobacteria. Among these, Firmicutes and Bacteroidetes were dominant, contributing respectively 73.3% and 23.4% in the NC group, 80.8% and 18.2% in the PC group, and 80.3% and 18.2% in the 10RJ group (Fig. 3). In the NC group, 1.8% of the organisms were Proteobacteria compared to 0.2% and 0.4% for PC and 10RJ (*P* > 0.05). Actinobacteria and Tenericutes ranged from 0.1% to 1.1% in all groups (*P* > 0.05). The abundance of Firmicutes was greater (*P* > 0.05) in the PC and 10RJ groups than in the NC group while that of Bacteroidetes, Proteobacteria and Tenericutes was lower. Increases in fecal Firmicutes appear to be related to nutrient absorption, whereas increases in Bacteroidetes have been associated with decreased nutrient absorption [32].

**Fig. 4:**
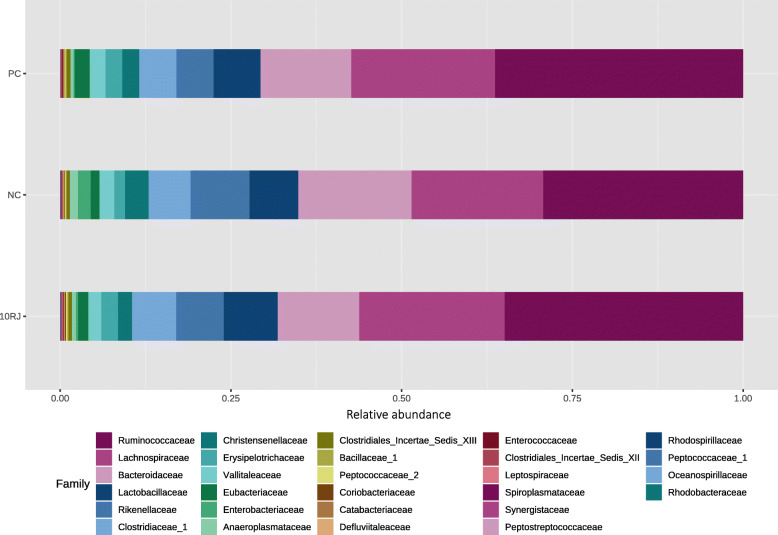
Percentage of relative abundance for the different treatments at taxonomical level family in the cecal digesta of broilers. Each mean represents eight samples. PC = basal diet with antibiotic bacitracin, NC = basal diet without antibiotic, 10RJ = basal diet with 5 mmol/L reuterin and 0.08 μmol/L J25 in drinking water

Rikenellaceae was more abundant in the NC group, whereas Ruminococcaceae and to a lesser extent Lachnospiraceae were favored in the PC and 10RJ groups (Fig. 4). The 10RJ treatment decreased the abundance of Bacteroidaceae compared to NC. Ruminococcaceae, recognized as a late colonizer of the chicken cecum, and Lachnospiraceae, both major representatives of the Firmicutes in the cecum, were more abundant in the PC and 10RJ groups. Ruminococcaceae is more abundant in birds with low feed conversion ratios [33], which is consistent with the present study. Both families are also associated with gut health through degradation of plant materials [34]. The responses obtained in association with these families confirm the impact of the intestinal microbiota on feed digestion and assimilation. Species of the family Bacteroidaceae have a large genome, which favors their adaptation to different environmental factors [17]. In our study, this family was affected by 10RJ and bacitracin, indicating their responsiveness to gut environmental conditions. Less abundant and not differing among the groups, Lactobacillaceae is notable for fermentative activities.

**Fig. 5:**
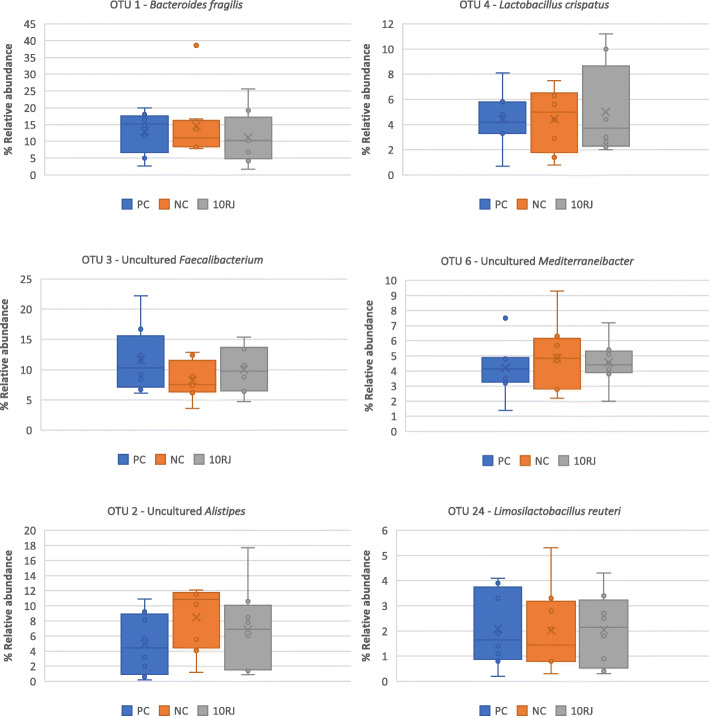
Boxplots of the relative abundance of the top 6 operational taxonomic units (OTUs) in the three treatments. PC = basal diet with antibiotic bacitracin, NC = basal diet without antibiotic, 10RJ = basal diet with 5 mmol/L reuterin and 0.08 μmol/L J25 in drinking water. *P* > 0.05

The ceca contained 2970 species-level OTUs, of which top 6 were identified more abundant than 2% each. Closely related to *Bacteroides fragilis*, OTU1 accounted for 14.6% in the NC group and 11.2% in the 10RJ group (Fig. 5). An uncultured *Faecalibacterium,* OTU3 made up 11.6% of the PC group microbiota, 10% in the 10RJ group and 8.2% in the NC group. Bacitracin and 10RJ both increased the predominance of the genus *Faecalibacterium* (*P* > 0.05) and decreased *Bacteroides* (*P* > 0.05) compared to NC. OTU2, assigned to an uncultured *Alistipes,* was the most abundant genus (8.5%) in the NC group (*P* > 0.05). The genus *Lactobacillus* was represented mainly by OTU4 (*Lactobacillus crispatus*) and OTU24 (*Limosilactobacillus reuteri*), which varied little (4.4–5% and 2–2.1% respectively; *P* > 0.05). OUT6 was assigned to an uncultured *Mediterraneibacter* (4.6–4.8%). The predominant *Bacteroides* species in broiler fecal microbiota has been identified as *B. fragilis*, at 45.3% [35]. This is also the most commonly isolated anaerobic pathogen, due in part to its potent virulence factors [36]. In contrast, *Lactobacillus* and *Faecalibacterium* are known as health-promoting bacteria. Since *Alistipes* is a relatively recent sub-branch genus of the Bacteroidetes phylum and Bacteroidetes are commonly associated with chronic human intestinal inflammation [37], the 10RJ treatment could reduce the risk of contamination by such pathogens in the cecum and thus contribute to maintaining good carcass quality.

**Fig. 6:**
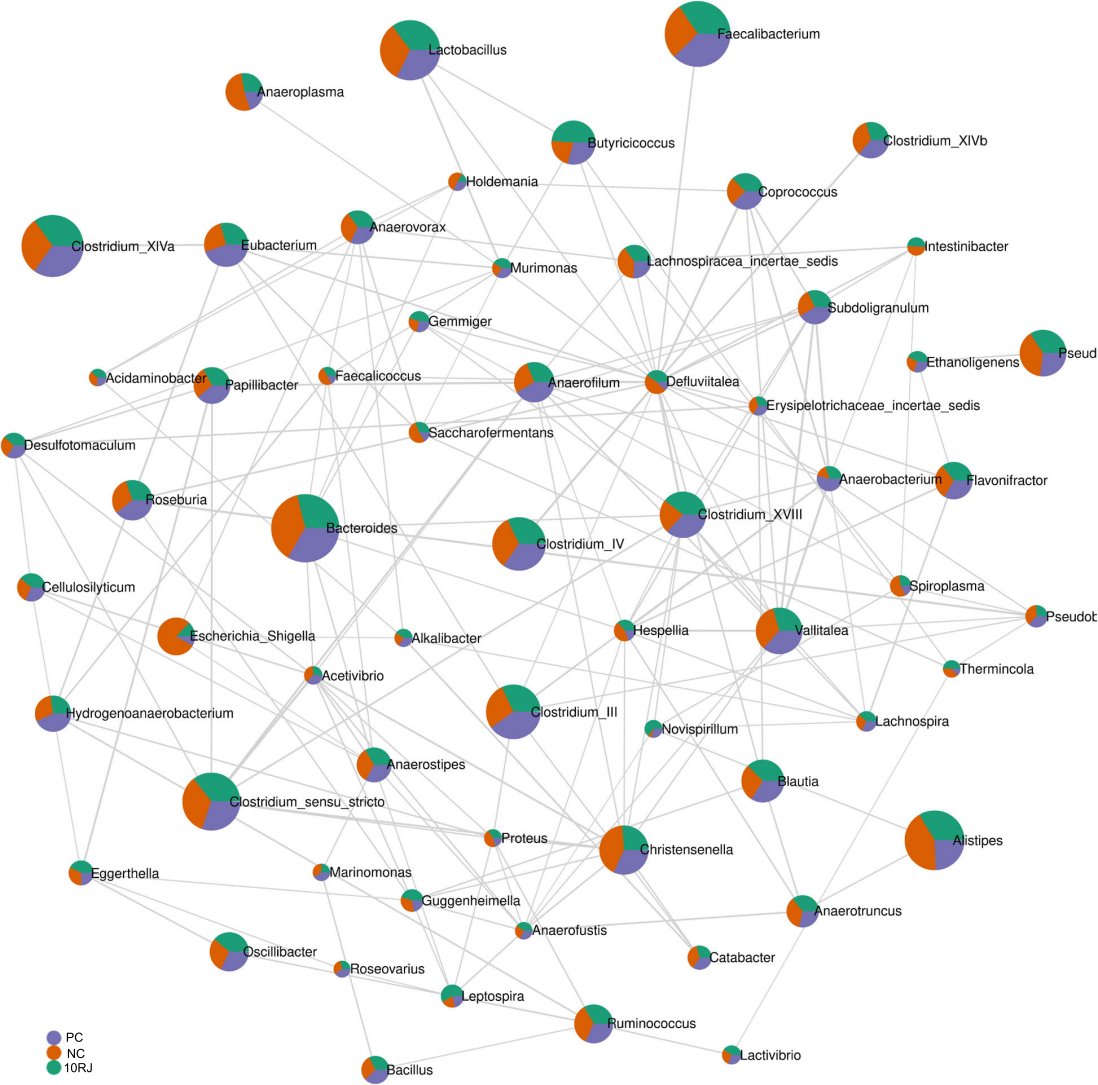
Microbial network at genus level based on correlation analysis. PC = basal diet with antibiotic bacitracin, NC = basal diet without antibiotic, 10RJ = basal diet with 5 mmol/L reuterin and 0.08 μmol/L J25 in drinking water

Connectivity was investigated using network analysis, with the components restricted to the genus level (Fig. 6). Co-occurrence patterns are used to depict the co-existence and maintenance of the microbes in a determined environment, the idea being that interactions are stronger in more stable communities [17]. It is interesting that *Lactobacillus* and *Bacteroides* behave like keystone genera, since they are detected at higher abundance and with greater connectivity. Furthermore, there is no direct connection between highly abundant genera such as *Bacteroides, Faecalibacterium, Clostridium* XIVa, *Lactobacillus* and *Alistipes*, indicating that changes in the main intestinal bacterial species require interaction of the entire ecosystem.

It is well known that gut microbes make up a complex ecosystem that has a symbiotic relationship with the host and plays an important role in the immune system. Changes in gut microbiota composition alter the complement of genes expressed for reinforcing specific host metabolic pathways [1]. While the use of natural antimicrobials to modify gastrointestinal microbiota and thereby maintain animal health is considered valid, whether or not this will be the case for RJ remains to be confirmed, with very limited researches so far.

### Microbial metabolites in the cecal digesta

**Fig. 7:**
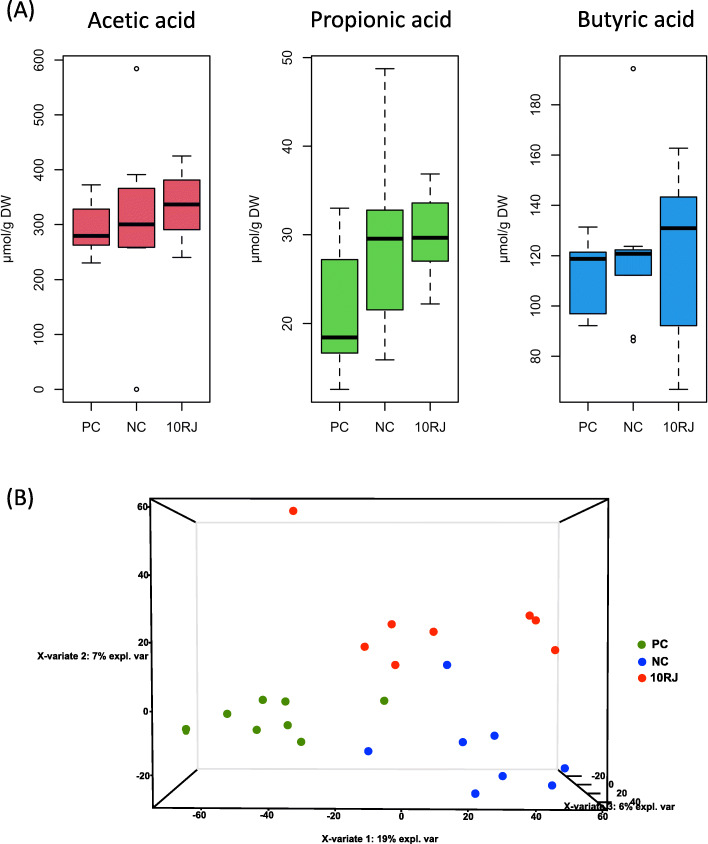
(**A**) The short-chain fatty acid concentrations in the cecal content (*n* = 8 for each group; *P* > 0.05); (**B**) PLS-DA plot for the total cecal metabolites in broiler gut. PC = basal diet with antibiotic bacitracin, NC = basal diet without antibiotic, 10RJ = basal diet with 5 mmol/L reuterin and 0.08 μmol/L J25 in drinking water

Bacitracin and 10RJ have different impact on the microbial metabolites, as shown in Fig. 7, although with an important variability. To compensate for variable dilution of the cecal contents by water, the samples were adjusted to a standard dry mass per mL and the SCFA concentrations were normalized to dry mass. Analyte stability is of special importance for metabolically highly active specimens. By transporting samples on dry ice, we tried to avoid the changes in SCFA concentration that may occur within a few hours at room temperature [38]. Interestingly, freezing of samples and subsequent storage at room temperature has been shown to result in decreased SCFA concentrations [24]. Huge differences between experiments can be due to changes that occur during sample storage.

In the 10RJ group, acetic, propionic, and butyric acids were all increased compared to the NC group, while the bacitracin group was unexpectedly different, in which the concentrations of all SCFAs were lower than that of the NC group (Fig. 7A). It therefore appears likely that 10RJ has a different influence on the microbiome metabolic pathways.

The microbial metabolite profiles in the intestinal digesta reflect microbial activity and gut health. This is the first investigation of changes to the cecal metabolome in growing broilers given RJ. SCFAs are the principal end products of carbohydrate fermentation by gut microbes and serve as indicators of microbial activity. The present increases in SCFA concentrations suggest that this antimicrobial combination enhances carbohydrate fermentation by bacteria. Ruminococcaceae including *Faecalibacterium* are major producers of butyrate [39], and *Lactobacillus* is also deeply involved in carbohydrate fermentation. The increases in SCFAs were associated with greater prevalence of these beneficial bacteria. Acetate can be used as an energy substrate in many peripheral tissues, butyrate is the main energy source of colonic epithelial cells and has an anti-inflammatory effect, and lactate can lower the pH in the gastrointestinal tract and inhibit the multiplication of pathogens such as *E. coli*, which otherwise invade the gut [25]. These increases of SCFA concentrations suggest that the 10RJ treatment creates a host-friendly gut environment.

As modifiers of the intestinal microbiota, antibiotics affect the microbial metabolism of carbohydrates and amino acids in the cecum, leading to decreases in the concentrations of lactate and most SCFAs and increases in the concentrations of branched-chain fatty acids, many amines and *p*-cresol [25]. Our present results are consistent with this and with other previous studies [24, 25, 40–42] in which antibiotic treatments led to a decrease in most SCFA concentrations (less carbohydrate fermentation) and increased amine concentrations (more microbial decarboxylation of amino acids). Overall, our present findings provide clear evidence of a shift in microbial metabolic activity in the broiler cecum following treatment with bacitracin or with 10RJ.

The PLS-DA plot confirmed that the treatment effects on cecal metabolites were distinct from each other (Fig. 7B), further supporting that bacitracin and 10RJ altered the broiler intestinal microbiota. However, wide variability within groups was also observed. 10RJ was shown to improve the microbiota and regulate gut microbiome metabolism directly. Antibiotics are usually considered the most cost-effective way to reduce pathogenic bacteria and modify the gut environment. However, they have been shown recently to cause gut microbiota dysbiosis, inhibit innate immune defenses and thereby increase pathogen colonization and disease susceptibility [25]. Compared to bacitracin, 10RJ therefore could be viewed as more beneficial to poultry intestinal health and overall performance and less of a burden on the environment.

Profiles of free amino acids and their metabolites might also need to be studied in association with bacitracin or 10RJ treatment. Broilers fed the control diet may have spent more energy generating certain amino acids by endogenous metabolism rather than obtaining some portion of them via microbial fermentation by the beneficial bacteria favored by these antimicrobial treatments. Comparison of serum metabolomes has shown that obese pigs develop distinctive protein and amino acid metabolism compared to lean pigs [43]. Furthermore, such studies have shown that supplementing the diet of weaned piglets with amino acids can alter multiple metabolic pathways associated with lipid metabolism [44]. Protein and lipid metabolic pathways therefore need to be investigated for complete analysis of changes to broiler cecal contents.

Other studies have shown that the composition and profile of the intestinal microbiota and metabolites thereof continually change in individuals [1, 45]. The high-throughput sequencing data certainly showed that bacitracin and 10RJ changed the cecal microbiota in broilers in the grower period after treatment. The metabolomic analysis also revealed different treatment effects within 3 weeks. However, the microbial similarity and metabolic differences between the bacitracin and 10RJ treatments indicate that the predominant gut community might not be correlated directly with metabolites. The metabolic pathways involved form a very complex system that includes the tricarboxylic acid cycle, fatty acid synthesis and metabolism of lipids, amino acids and so on. More comprehensive metabolomic analyses will be needed for future studies.

## Conclusion

In summary, the present study demonstrated that the 10RJ added to drinking water could be used to achieve the effect of improving the growth performance of broilers, which was obtained by the conventional use of antibiotics as growth promoters. Moreover, this non-antibiotic combination promoted the abundance of certain bacterial species in a manner similar to antibiotic, thereby changing the cecal bacterial community. It was associated with increased abundance of well-recognized beneficial microorganisms belonging to Lachnospiraceae*,* Ruminococcaceae and Lactobacillaceae. Furthermore, this combination altered the microbial metabolome, at least in terms of the carbohydrates. However, the regulation of protein and lipid metabolism remains to be investigated to reveal the full complexity of the intestinal metabolic processes in broiler. Overall, these findings suggest that 10RJ may be a suitable replacement for antibiotics in broiler chicken production.

## Supplementary Information

**Additional file 1: Supplementary Table 1.** Composition of the basal diet fed to broiler chickens in different phases of trial for 35 d. **Supplementary Table 2.** Temperature program. **Supplementary Table 3.** Lighting schedule. **Supplementary Table 4.** Retention times and *m/z* ratios of the 3-NPH SCFA derivatives and corresponding stable isotope labelled internal standards. **Supplementary Fig. 1.** Daily recorded humidity and temperature

## Data Availability

The datasets used and/or analysed during the current study are available from the corresponding author on reasonable request.

## Abbreviations

SCFA: Short-chain fatty acids
OTUs: Operational taxonomic units
3-NPH: 3-Nitrophenylhydrazone
PCoA: Principal coordinate analysis
