# Supplementary material for: Effects of drinking water supplementation with Lactobacillus reuteri, and a mixture of reuterin and microcin J25 on the growth performance, caecal microbiota and selected metabolites of broiler chickens
Source: J Anim Sci Biotechnol. 2022 Mar 5;13:34. doi: 10.1186/s40104-022-00683-6 (PMC8897850; doi:10.1186/s40104-022-00683-6)
Supplement: Supplementary file 1 — Additional file 1: Supplementary Table 1. Composition of the basal diet fed to broiler chickens in different phases of trial for 35 d. Supplementary Table 2. Temperature program. Supplementary Table 3. Lighting schedule. Supplementary Table 4. Retention times and m/z ratios of the 3-NPH SCFA derivatives and corresponding stable isotope labelled internal standards. Supplementary Fig. 1. Daily recorded humidity and temperature [file 40104_2022_683_MOESM1_ESM.docx]

**Additional file**

**Supplementary Table 1**. Composition of the basal diet fed to broiler chickens in different phases of trial for 35 d.

| **Ingredient, %** | **Starter (0–10 d)** | | | **Grower (11–22 d)** | | | **Finisher (23–35 d)** | | |
| --- | --- | --- | --- | --- | --- | --- | --- | --- | --- |
|  | Calculated | Analyzed | | Calculated | Analyzed | | Calculated | Analyzed | |
|  |  | PC ^a^ | NC ^b^ |  | PC | NC |  | PC | NC |
| Crude protein | 22.0 | 22.5 | 22.6 | 20.0 | 19.9 | 21.1 | 18.0 | 17.9 | 18.8 |
| Crude fat | 3.35 | 3.10 | 3.20 | 4.32 | 4.50 | 4.30 | 6.37 | 6.50 | 6.20 |
| Total calcium | 0.82 | 0.80 | 0.84 | 0.72 | 0.66 | 0.70 | 0.67 | 0.66 | 0.73 |
| Total phosphorus | 0.59 | 0.58 | 0.63 | 0.53 | 0.52 | 0.54 | 0.47 | 0.47 | 0.50 |
| Total sodium | 0.18 | 0.17 | 0.18 | 0.17 | 0.16 | 0.16 | 0.16 | 0.18 | 0.17 |
| Chloride | 0.20 | 0.20 | 0.23 | 0.21 | 0.21 | 0.21 | 0.23 | 0.21 | 0.23 |
| Total magnesium | 0.17 | 0.15 | 0.16 | 0.16 | 0.15 | 0.15 | 0.14 | 0.14 | 0.14 |

^a^ Positive control diet with antibiotic; ^b^ Negative control diet without antibiotic

**Supplementary Table 2.** Temperature program

| **Days** | **Temperature, ^o^C** | **Temperature, ^o^F** |
| --- | --- | --- |
| 0 | 33.0 | 91.4 |
| 1 | 32.7 | 90.8 |
| 5 | 31.3 | 88.3 |
| 10 | 29.5 | 85.2 |
| 15 | 27.8 | 82.0 |
| 20 | 26.1 | 78.9 |
| 25 | 24.3 | 75.8 |
| 30 | 22.6 | 72.7 |
| 35 | 20.5 | 68.9 |

**Supplementary Table 3.** Lighting schedule

| **Days** | **Hours of light** | **Hours of darkness ^*^** | **Light intensity, lux** |
| --- | --- | --- | --- |
| 0 | 24 | 0 | 20 |
| 1 | 23 | 1 | 20 |
| 2–3 | 20 | 4 | 20 |
| 4–10 | 20 | 4 | 10 |
| 11–25 | 18 | 6 | 5 |
| 26–35 | 20 | 4 | 5 |

^*^ Darkness started at 9 PM every day.

**Supplementary Table 4**. Retention times and *m/z* ratios of the 3-NPH SCFA derivatives and corresponding stable isotope labelled internal standards.

| **3-NPH SCFA** | **Retention time, min** | ***m/z*** |
| --- | --- | --- |
| Acetic acid | 2.8 | 194.06 |
| [D_3_] acetic acid | 2.8 | 197.08 |
| Propionic acid | 3.5 | 208.07 |
| [D_5_] propionic acid | 3.5 | 213.10 |
| Butyric acid | 4.2 | 222.09 |
| [D_7_] butyric acid | 4.2 | 229.13 |

**Supplementary Fig. 1.** Daily recorded humidity and temperature
